# Aminoglycosides rapidly inhibit NAD(P)H metabolism increasing reactive oxygen species and cochlear cell demise

**DOI:** 10.1117/1.JBO.24.5.051403

**Published:** 2018-11-08

**Authors:** Danielle E. Desa, Michael G. Nichols, Heather Jensen Smith

**Affiliations:** a University of Rochester, Department of Biomedical Engineering, Rochester, New York, United States; b Creighton University, Department of Physics, Omaha, Nebraska, United States; c Creighton University, Department of Biomedical Sciences, Omaha, Nebraska, United States; d University of Nebraska Medical Center, The Eppley Institute for Cancer and Allied Diseases, Omaha, Nebraska, United States

**Keywords:** aminoglycoside, ototoxicity, hearing loss, metabolic imaging, nicotinamide adenine dinucleotide, reactive oxygen species

## Abstract

Despite causing permanent hearing loss by damaging inner ear sensory cells, aminoglycosides (AGs) remain one of the most widely used classes of antibiotics in the world. Although the mechanisms of cochlear sensory cell damage are not fully known, reactive oxygen species (ROS) are clearly implicated. Mitochondrial-specific ROS formation was evaluated in acutely cultured murine cochlear explants exposed to gentamicin (GM), a representative ototoxic AG antibiotic. Superoxide (${{O_{2}}^{\cdot }}^{-}$) and hydrogen peroxide ($H_{2}O_{2}$) were measured using MitoSOX Red and Dihydrorhodamine 123, respectively, in sensory and supporting cells. A 1-h GM exposure significantly increased ${{O_{2}}^{\cdot }}^{-}$ formation in IHCs and increased $H_{2}O_{2}$ formation in all cell types. At the same time point, GM significantly increased manganese superoxide dismutase (MnSOD) levels while significantly decreasing copper/zinc superoxide dismutase (CuZnSOD) in cochlear sensory cells. This suggests (1) a rapid conversion of highly reactive ${{O_{2}}^{\cdot }}^{-}$ to $H_{2}O_{2}$ during the acute stage of ototoxic antibiotic exposure and (2) that the endogenous antioxidant system is significantly altered by AGs. Fluorescence intensity-based measurements of reduced nicotinamide adenine dinucleotide (phosphate) [NAD(P)H] and mitochondrial membrane potential were measured to determine if increases in GM-induced ROS production were correlated with changes in mitochondrial metabolism. This project provides a basis for understanding the mechanisms of mitochondrial ROS production in cochlear cells exposed to ototoxic antibiotics. Understanding the nature of ototoxic antibiotic-induced changes in mitochondrial metabolism is critical for developing hearing loss treatment and prevention strategies.

## 1 Introduction

Aminoglycoside (AG) antibiotics are widely used for the treatment of gram-negative bacterial infections. Though they are highly effective and relatively cheap, AGs are known to be both oto- and vestibulotoxic, affecting the auditory and vestibular systems of the inner ear, respectively. All AGs in use globally show some degree of ototoxicity and vestibulotoxicity,^1^ causing permanent hearing and/or balance disorders by damaging auditory and vestibular sensory cells. As ototoxicity progresses, so too does the extent of permanent hearing loss as mammalian cochlear and vestibular sensory cells are unable to regenerate. Consequently, there is a long-standing need to understand the mechanism(s) by which AGs cause irreversible cell damage and to develop strategies to mitigate these consequences when using life-saving antibiotics.

The cochlea contains sensory inner and outer hair cells (IHCs and OHCs), responsible for transducing sound waves into nerve signals and the active amplification of acoustic signals, respectively. Cochlear sensory cells are interdigitated and structurally reinforced by several types of supporting cells, including pillar and Deiters cells. The cochlea is additionally tonotopically organized along its length; higher frequency sounds are processed in the basal turn, whereas lower frequency sounds are processed in the apical turn. Although all mammalian cells are traditionally considered to be impervious to antibiotics, cationic AGs have been shown to selectively gain entry into cochlear sensory cells (I/OHCs) via highly specialized mechanotransduction channels located in the apical stereociliary bundle.^2^ Furthermore, others have suggested the potent electrophoretic force generated by the charge differential between the cochlear cell-surrounding endolymph (+80  mV) and cochlear hair cell receptor potential (−70  mV) drives positively charged AGs into cochlear sensory cells through nonselective cation channels.^3^ Accordingly, polarized cochlear sensory cells are indeed preferentially damaged by AGs, with high-frequency OHCs exhibiting the greatest susceptibility to AG-induced damage. With prolonged AG exposure, injury progresses to low-frequency regions of the cochlea and may result in total hearing loss triggered by sensory cell death.^4^ AGs inhibit bacterial protein synthesis by binding to the A-translational site of 16S rRNA of the 30S ribosomal subunit, causing increased mRNA misreading.^5^^–^^9^ Less is known about the molecular mechanism(s) regulating AG-induced ototoxicity, nor why high-frequency OHCs are particularly susceptible. There is, however, mounting evidence that hair cell death may be the result of changes in mitochondrial metabolism, triggering overproduction of cell-damaging reactive oxygen species (ROS), ultimately leading to cell death. Several studies have shown a strong mitochondrial association with AG-induced sensory cell death and subsequent hearing loss including; increased AG-induced hearing loss with mitochondrial mutations,^10^^–^^13^ hypothesized AG-induced alterations in mitochondrial protein synthesis,^13^ and AG-induced sensory cell death has been shown to be triggered by mitochondrial calcium uptake.^14^

Although ROS are (1) a natural byproduct of normal cellular metabolism and (2) are responsible, when at low levels, for maintaining signaling pathways and homeostasis, increased ROS may overwhelm cellular antioxidant defenses, resulting in oxidative stress/cellular damage and/or death.^15^ Indeed, numerous studies have established ROS as key mediators of cochlear sensory cell damage in AG-, age-, and noise-induced hearing loss.^16^^–^^19^ Despite excessive ROS production being a well-characterized feature of AG ototoxicity, the source of ROS remains poorly understood. The studies contained herein seek to extend recent reports indicating alterations in mitochondrial metabolism, including possible inhibition of mitochondrial respiratory complexes involved in oxidative phosphorylation, may be responsible for AG-induced cochlear sensory cell and hearing loss.

Notably, endogenous nicotinamide adenine dinucleotide (NADH) fluorescence can be used as an indicator of mitochondrial energy metabolism in living cells. Resting levels of NADH represent a balance of NADH production, primarily by glycolysis and the tricarboxylic acid (TCA) cycle, and NADH consumption via oxidation by NADH dehydrogenase in the electron transport chain (ETC). While this leads to the generation of adenosine triphosphate (ATP), it is also the most significant source of endogenous free radicals (ROS). NADPH is similarly involved in a number of processes, including maintenance of reduced glutathione levels for antioxidant defense. Though NADH and NADPH fluorescence are spectrally indistinguishable, previous studies manipulating cochlear cell NAD(P)H fluorescence intensity with sodium cyanide and the metabolic uncoupler carbonylcyanide-4-(trifluoromethoxy)-phenylhydrazone (FCCP), which specifically affect mitochondrial oxidative metabolism, have shown cochlear NAD(P)H fluorescence is predominated by NADH fluorescence.^20^^,^^21^ Measurements of endogenous NAD(P)H fluorescence intensity and lifetimes in cochlear sensory and supporting cells revealed endogenous differences in I/OHC and supporting cell mitochondrial metabolism.^22^ Furthermore, high-frequency OHCs preferentially respond rapidly (0.5 to 1 h) to changes in their microenvironment, including increased glucose levels and AG exposure.^19^ When NAD(P)H intensities are coupled with fluorescence lifetime imaging (FLIM), free and various protein-bound forms of NAD(P)H may be distinguished. Acute (0.5 h) AG treatment increases NAD(P)H lifetimes in low-frequency IHCs, as well as NAD(P)H concentration in high-frequency OHCs. These changes are consistent with a shift from free to enzyme-bound NADH. Furthermore, these changes have been observed within 0.5 h of AG exposure, long before mitochondrial protein synthesis would be inhibited. Hence, we propose that AG-induced ototoxicity is the result of changes to mitochondrial metabolism specifically occurring in cochlear sensory cells during the acute stages (≤24  h) of AG exposure.^22^

The studies described in the present work investigate the rapid (≤24  h) impact of AG exposure on mitochondrial metabolism in both high- and low-frequency sensory and supporting cells of the cochlea. In addition to measuring changes in metabolism, these studies describe downstream effects including mitochondrial ROS formation and decreased antioxidant levels occurring within the initial hour of AG exposure. Finally, our assessments of increased mitochondrial-mediated pro-apoptotic pathway activity during acute AG exposures suggest a mechanism for rapid cellular compromise in response to AGs. These studies provide a basis for understanding the mechanism(s) by which cochlear metabolic dysfunction occurs and leads to cochlear cell death and hearing loss.

## 2 Materials and Methods

### 2.1 Cochlear Explant Imaging

All live-cell imaging studies were performed using acutely cultured, intact, organotypic cochlear explants obtained from postnatal day 6 (P6±1 d) FVB mice. Briefly, dissections were performed in HEPES-buffered L-15 medium (Life Technologies, Carlsbad, California) where Reissner’s membrane was peeled away from intact strips of the cochlear basilar membrane to allow visualization of the organ of Corti. Intact, cochlear explants were incubated in Dulbecco’s modified Eagle Medium L-Glutamine/F12 medium (DMEM, Life Technologies, Carlsbad, California) supplemented with 15% fetal bovine serum (FBS), 1.5  μg/mL Amphotericin B (Gibco) and 150  U/mL penicillin and maintained at 37°C and 5% ${\mathrm{CO}}_{2}$ for 10 to 16 h prior to experimentation. Cochlear explants showing no overt signs of mechanical trauma or cellular damage were subsequently exposed to GM for different amounts of time (0.5, 1, 3, 12, and 24 h) then identically loaded with individual fluorescent indicators, as described below. Samples requiring fixation prior to labeling were time-matched (∼15  min Tyrodes rinse) to live cochlear explant exposures to optimize the temporal resolution across measurements. Due to its low cost and consistent bactericidal activity, GM is one of the most commonly used AGs in the clinic despite its association with hearing loss.^3^^,^^23^ As such, GM was chosen as a representative AG antibiotic. All experiments used GM at 300  μg/mL (521  μM), well within the range of doses (0.1 to 1 mM) used by others to describe GM-induced cochlear insults.^24^^,^^25^ Finally, while others have described preferential GM-uptake, and cellular loss in high-relative to low-frequency sensory cells,^24^^–^^26^ we selected a dose previously shown by our group to display similar GM accumulation in high- and low-frequency cells.^22^

**Fig. 1:**
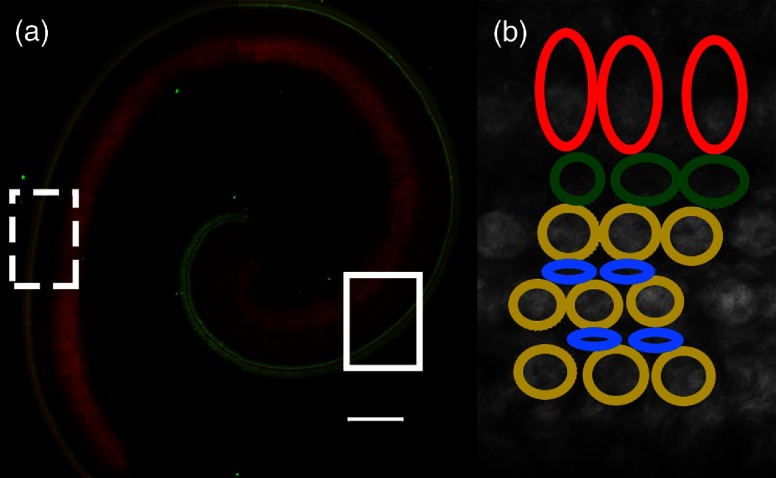
Organization of the sensory apparatus of the mammalian cochlea, the organ of Corti. (a) The cochlea and organ of Corti are tonotopically organized; high-frequency sounds are processed in the basal turn (dashed box), while low-frequency sounds are processed in the apical turn (solid box). Green = Myosin7a-labeled sensory cells, red = F-actin. Scale bar=200  μm. (b) Representative fluorescence intensity image (DHR123) showing the relative location of cochlear sensory (IHC, red; OHC, yellow) and supporting (pillar, green; Deiters, blue) cells. Scale bar=25  μm.

**Fig. 2:**
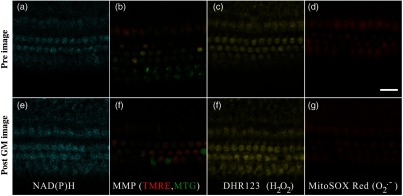
Representative fluorescence intensities of various metabolic and ROS indicators in cochlear sensory and supporting cells. (a, e) Endogenously fluorescent NADH was observed in representative cochlear explants before and after 0.5-h GM exposure. (b, f) MMP was calculated as the ratio of TMRE/MTG fluorescence intensities before and after 0.5-h GM exposure (representative images), respectively. (c, g) The mitochondrial-specific $H_{2}O_{2}$ indicator, DHR123, was measured before and after 1-h GM exposure (representative images), respectively. (d, h) The mitochondria-specific ${{O_{2}}^{\cdot }}^{-}$ indicator, MitoSOX Red, was measured before and after 0.5-h GM exposure (representative images). Scale bar=25  μm.

Sensory and supporting cells were imaged in the high-frequency basal turn and in the low-frequency apical turn of each sample [Fig. 1(a)]. Explants were maintained at 32°C±1°C during imaging using a warmed platform and temperature controller throughout imaging (Warner Instruments, Hamden, Connecticut). For all live cell imaging experiments, images were acquired at a 600 Hz line scan rate resulting in a frame time of 2.4 s. Murine cochlear explants, 300 to 700  μm in total thickness (z-coordinate), contain sensory and supporting cells <150  μm from the surface. Notably, cochlear sensory cells vary in length along the tonotopically organized cochlea such that basal turn, high-frequency sensory cells are ∼15  μm in length, while apical turn, low-frequency sensory cells are ∼25  μm in length. Cochlear sensory and supporting cells reside on the apical surface of cochlear explants. Images (3-μm focal volume/image) of endogenous and exogenous fluorophores were collected using a 3-μm (z-coordinate) sequential focal step throughout each explant. To ensure all cells in a given image window (see Fig. 2) were entirely imaged, z coordinates for initiating and ending whole explant imaging regularly included 1 to 2 images above and/or below each group of analyzed cells. Image stacks consisting of a total size of 7 to 15 images, totaling 21 to 45  μm in the z-coordinate, were acquired with an average acquisition time of ∼36  s.^19^^,^^21^^,^^22^

Each analyzed image window measured ∼88×88  μm and contained ∼32 sensory (∼8 IHC, ∼24 OHC) and 16 supporting (∼8 pillar and ∼8 Deiters) cells. Figure 1(b) shows the organization of the organ of Corti, including the relative location of cochlear sensory (I/OHCs) and supporting (pillar and Deiters) cells. As represented for a restricted subset of cells in Fig. 1(b), regions of interest (ROIs) were manually drawn around individual cells in each image, propagated through the image stack until individual cells were no longer observed, then analyzed using ImageJ. To control for differences in length between high- and low-frequency cells, mean fluorescence intensities (endogenous and exogenous fluorophores) for individual cells were determined by averaging the cell/individual ROI fluorescence intensities obtained from each image in the image stack.^27^^,^^28^

All animal care and use procedures were approved by the Creighton University Animal Care and Use Committee.

### 2.2 Determination of NADH Fluorescence Intensity

To assess NADH fluorescence intensity, cochleae were incubated in DMEM with GM for various amounts of time at 37°C and 5% ${\mathrm{CO}}_{2}$. Prior to imaging, both GM-exposed and time-matched control samples were placed in a modified Tyrode’s imaging buffer (135 mM NaCl, 5 mM KCl, 1 mM ${\mathrm{MgCl}}_{2}\cdot 6H_{2}O$, 1.8 mM ${\mathrm{CaCl}}_{2}\cdot 2H_{2}0$, 20 mM HEPES, 5 mM glucose, and 0.25% bovine serum albumin) for 0.5 h at 37°C. Fluorescence intensity imaging of two-photon-excited NADH was subsequently performed using the 740-nm mode-locked pulse train of a Spectra Physics Mai Tai Ti:S laser and a HCX IRAPO 25× 0.95 NA water immersion objective on a Leica TCS SP8 MP multiphoton laser scanning confocal microscope (Leica Microsystems, Buffalo Grove, Illinois). The average power at the sample was ∼1.2  mW. The nondescanned fluorescence from NADH was isolated using a 500-nm long-pass dichroic mirror and a HQ 460/80 band-pass filter (Chroma Technology, Bellows Falls, Vermont) and detected with high-sensitivity super HyD detectors [Figs. 2(a) and 2(e)].

### 2.3 Measurement of Mitochondrial Membrane Potential

The mitochondrial membrane potential (MMP) is an essential component of the proton motive force that contributes to ATP production. Therefore, changes in MMP may indicate metabolic dysfunction. To assess changes in MMP, control and GM-exposed cochlear explants were incubated with 250  μM tetramethylrhodamine-ethyl-ester-perchlorate (TMRE), a fluorescent MMP indicator, and 200 nM MitoTracker Green (MTG), a membrane potential-independent fluorescent mitochondrial label, at 37°C and 5% ${\mathrm{CO}}_{2}$ for 30 and 20 min, respectively. TMRE and MTG were single-photon excited using 552- and 488-nm excitation with collection at 565 to 620 nm and 500 to 550 nm, respectively [Figs. 2(b) and 2(f)]. Relative MMP differences were calculated as the ratio of TMRE/MTG average fluorescent intensities from each cell type and each treatment condition.^22^

### 2.4 Measurement of Mitochondrial-Generated ROS

To assess mitochondrial-specific ROS levels, cochlear explants were exposed to Dihydrorhodamine 123 (DHR123) or MitoSOX Red to assess hydrogen peroxide ($H_{2}O_{2}$) or superoxide (${{O_{2}}^{\cdot }}^{-}$) levels, respectively. MitoSOX Red is positively charged and accumulates in the mitochondrial matrix, where it can be specifically oxidized by ${{O_{2}}^{\cdot }}^{-}$, resulting in detectable fluorescence. Cochlear explants were incubated with GM for 0.5 to 24 h at 37°C and 5% ${\mathrm{CO}}_{2}$. Prior to imaging, both GM-exposed and time-matched control samples were rinsed with Hank’s buffered salt solution (HBSS), incubated (20 min) in 5  μM MitoSOX Red or 200 nM DHR123, then rinsed and maintained in Tyrodes buffer during imaging. In separate studies, DHR123 and MitoSOX Red were excited at 514 nm with the resulting fluorescence emission filtered using either a 525 to 595 [DHR123, Figs. 2(c) and 2(g)] or 560 to 620 nm [MitoSOX Red, Figs. 2(d) and 2(h)] spectral bandpass filter and subsequent detection by an internal HyD detector.

The mean fluorescence intensity from each cell type was measured using ImageJ as previously described. Although DHR123 loading and resulting fluorescence intensities were similar across preparations, fluorescence intensity from GM-exposed samples was corrected by subtracting the mean DHR123 intensity obtained from time-matched controls prepared and imaged on the same day at the same time points (representing endogenous, baseline ROS produced by cellular metabolism). Similarly, MitoSOX Red intensity was corrected using an average intensity measurement from multiple control and apical IHC samples. The average value for control, apical IHCs in each experiment was divided by this value to produce a correction factor. All treatment groups’ intensity values were multiplied by this correction factor to account for day-to-day system and biological loading variability, as well as endogenous baseline ROS levels.

### 2.5 NADH Dehydrogenase-Specific ROS Measurements

Rotenone (RTN) is an ETC complex I inhibitor that blocks the transfer of electrons from complex I iron–sulfur centers to ubiquinone, thereby decreasing oxidative phosphorylation and ATP production while increasing complex I-specific ROS levels.^29^ We hypothesized that GM would produce similar effects on ${{O_{2}}^{\cdot }}^{-}$ levels if it blocked respiratory complex I where RTN does. To assess complex I- (NADH dehydrogenase) specific ${{O_{2}}^{\cdot }}^{-}$ levels, MitoSOX Red fluorescence intensities were measured before and after RTN application and in the presence and absence of GM. A concentration series was performed to determine the minimum RTN concentration needed to significantly increase ROS without significantly altering MMP. About 250-nM RTN significantly increased ${{O_{2}}^{\cdot }}^{-}$ levels (MitoSOX Red fluorescence intensity) while minimally affecting MMP (TMRE/MTG, data not shown). About 250 nM RTN was subsequently applied to control and GM-exposed samples to assess the effects of RTN on GM-induced ${{O_{2}}^{\cdot }}^{-}$ levels (MitoSOX Red). As previously described, ROIs were manually drawn in ImageJ and fluorescence intensities for each cell type were corrected for day-to-day variability in cell loading.

### 2.6 Assessment of Mitochondrial and Cytoplasmic Superoxide Dismutases (SODs)

Acutely cultured cochlear explants were exposed to GM for 0.5 to 12 h, fixed in 10% formalin for 12 to 16 h, then stored in phosphate-buffered saline (PBS) before subsequent staining with primary antibodies directed against SOD1 (cytoplasmic CuZnSOD, Abcam ab16831, 1:100) and SOD2 (mitochondrial MnSOD, ThermoFisher, PA1-31072, 1:200). CuZnSOD and MnSOD antibodies were labeled using goat anti-rabbit conjugated with AlexaFluor 567.^30^^–^^32^ Fluorescently labeled secondary antibodies were excited using 552 nm and collected with a 600- to 650-nm detector bandpass and a HC PL APO CS2 63× 1.4 NA oil immersion objective. About 2.5-μm optical sections were collected at a line scan rate of 400 Hz with a line average of 2.

### 2.7 Release of Apoptosis-Inducing Factor Following Aminoglycoside Exposure

To assess apoptosis during GM exposure, samples exposed to GM for varying amounts of time were fixed in 10% formalin for 12 to 16 h, then stored in PBS before being stained with a primary antibody directed against apoptosis-inducing factor (AIF, Abcam, ab32516) and an AlexaFluor 488-conjugated secondary antibody. AlexaFluor 488 was excited using 488 nm and fluorescence collected with a 500- to 550-nm detector bandpass and a HC PL APO CS2 63× 1.4 NA oil immersion objective. Samples were also stained for F-actin using AlexaFluor 568 Phalloidin and 4′,6-diamidino-2-phenylindole (DAPI) to assess morphological changes in nuclei (Molecular Probes, Eugene, Oregon). AlexaFluor 568 was excited at 552 nm and emissions collected with a 580- to 646-nm detector bandpass, whereas DAPI was excited at 405 nm and emissions collected with a 406- to 459-nm detector bandpass and HyD detector.^33^^–^^35^

### 2.8 Statistical Analysis

Significant differences between high- and low-frequency regions, cell types, and treatment groups were assessed using one- and two-way ANOVAs and posthoc Student’s t-tests. Pearson’s pairwise correlation coefficients were calculated between all of the time course measurements described in Sec. 2.2–2.7 (DHR, MitoSOX, NADH, SOD1, SOD2, AIF, and % cell loss) for all cell types (IHC, OHC, pillar, and Deiters) and cochlear locations (apex and base), and represented in a correlation matrix.

## 3 Results

### 3.1 GM Induces Rapid Changes in Mitochondrial Metabolism

**Fig. 3:**
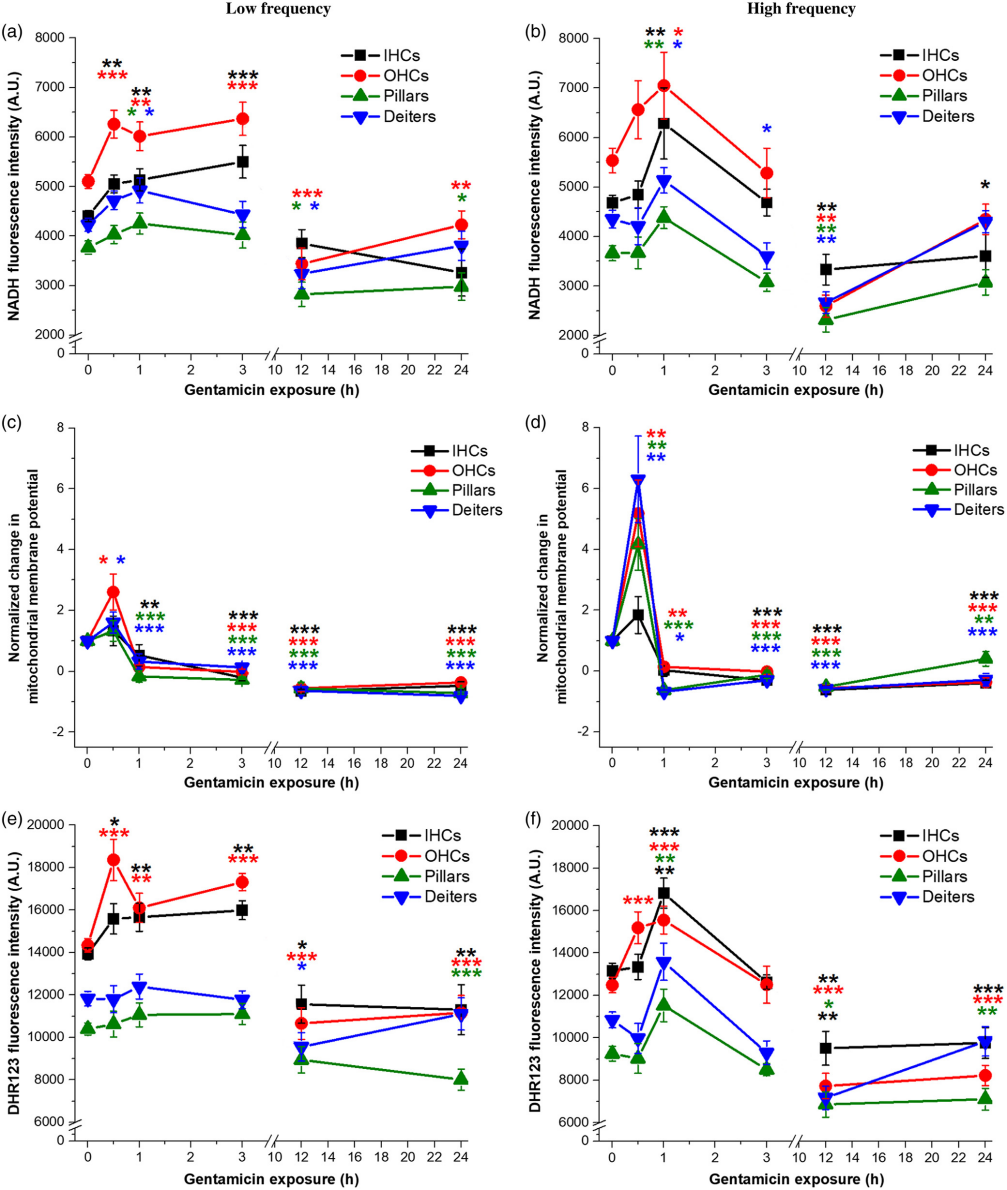
Acute GM exposure (≤24  h) alters cochlear cell mitochondrial metabolism and mitochondrial ROS production. (a) In low-frequency regions of the cochlea, a rapid, transient and significant increase in NADH fluorescence occurred, and persisted for 3 h in sensory cells. By 12 h, NADH was significantly below baseline in OHCs and supporting cells. (b) Akin to low-frequency regions, a transient increase in NADH was observed in high-frequency regions. (c) MMP rapidly increased (0.5 h), then decreased in OHCs and Deiters, then remained reduced in all low-frequency cells following acute GM. (d) High-frequency OHCs and supporting cells displayed a rapid (0.5 h) increase in MMPs and remained significantly reduced in all cell types by 3 h. (e) GM rapidly (0.5 to 3 h) increased $H_{2}O_{2}$ levels in low-frequency sensory cells. (f) In high-frequency cells, $H_{2}O_{2}$ levels rapidly increased in OHCs (0.5 h), increasing in all cells at 1 h and significantly decreasing in all cells by 12 h. Error bars = SEM. *p≤0.05, **p≤0.01, ***p≤0.001, relative to baseline.

To characterize acute AG-induced changes in mitochondrial metabolism, we first assessed NADH fluorescence intensity changes in individual cochlear cell types. Consistent with previous reports, NADH fluorescence intensities in sensory cells (I/OHCs) are greater than supporting (pillar, Deiters) cells, indicating fundamental differences in mitochondrial metabolism as seen in Figs. 2(a) and 2(e) and Figs. 3(a) and 3(b), respectively. We additionally observed metabolic changes during the acute phase (≤24  h) of GM exposure. Specifically, in low-frequency cochlear cells, we saw a significant increase in IHC (p≤0.01) and OHC (p≤0.001) NADH fluorescence intensity after 0.5-h GM exposure [Fig. 3(a)]. NADH levels remained significantly above baseline measurements for up to 3 h before decreasing. About 12-h post GM exposure, average NADH fluorescence intensities significantly decreased in OHCs (p≤0.001) and supporting cells (pillar, Deiters p≤0.05). At 24 h, NADH was significantly lower in OHCs (p≤0.01) and pillar cells (p≤0.05) but was not significantly different from baseline levels in IHCs and Deiters cells. In the high-frequency region [Fig. 3(b)], there was a significant increase in NADH fluorescence intensity in all cell types by 1 h (OHCs, pillar cells p≤0.01; IHCs, Deiters cells p≤0.05). By 3 h, NADH intensity was significantly lower in Deiters cells (p≤0.05) and by 12 h had decreased significantly in all cell types (p≤0.01). At 24 h, NADH levels remained significantly reduced in IHCs (p≤0.05) but returned to baseline in OHCs and supporting cells.

Mitochondria generate ATP by utilizing the electrochemical proton gradient, or MMP, established by the ETC across the inner mitochondrial membrane during oxidative phosphorylation. Given the significant changes observed in NADH following acute exposure to GM, we assessed MMP as another measure of putative metabolic dysfunction. TMRE fluorescence was used as a marker of the electrical MMP gradient, as previously described.^22^ TMRE fluorescence is sequestered in live mitochondria due to their highly negative charge, whereas inactive or depolarized mitochondria are unable to attract the positively charged dye and therefore are nonfluorescent. In low-frequency cochlear cells [Fig. 3(c)], MMP levels were significantly increased in OHCs and Deiters cells within 0.5 h of GM exposure (p≤0.05) but were subsequently decreased in IHCs (p≤0.01) and supporting cells (p≤0.001) after 1-h GM exposure. By 3 h, MMP levels dropped significantly below baseline in all cell types (p≤0.001) and this effect remained up to 24 h. In high-frequency cochlear cells [Fig. 3(d)], there was a rapid (0.5 h) and significant increase in MMP in OHCs (p≤0.01) and supporting cells (p≤0.01). MMP levels were significantly decreased in OHCs (p≤0.01), pillar (p≤0.001), and Deiters cells (p≤0.05) by 1 h. MMP fluorescence intensity was significantly reduced in all cell types by 3 h (p≤0.001) and remained significantly below baseline in all cell types 24-h post GM-exposure (p≤0.001).

### 3.2 GM Rapidly Increases ROS Levels in Cochlear Cells

Given that excess ROS levels are known to both stimulate, and be a consequence of subsequent metabolic perturbations, we used DHR123 to detect mitochondrial-specific changes in $H_{2}O_{2}$ levels over the same 24-h time course of GM exposure. As with NADH fluorescence intensity, there was a rapid (0.5 h) and significant increase in $H_{2}O_{2}$ in low-frequency sensory cells (p≤0.05 IHC, p≤0.001 OHC), which persisted for 3 h [Fig. 3(e)]. By 12 h, $H_{2}O_{2}$ levels decreased significantly in IHCs (p≤0.05), OHCs (p≤0.001), and Deiters cells (p≤0.05). At 24 h, $H_{2}O_{2}$ levels remained significantly decreased in IHCs (p≤0.01), OHCs (p≤0.001), and pillar cells (p≤0.001). In the high-frequency region [Fig. 3(f)], $H_{2}O_{2}$ levels significantly increased in OHCs within 0.5 h (p≤0.001) and in all cell types by 1 h (I/OHCs p≤0.001; pillar, Deiters cells p≤0.01). 12-h post GM-exposure, $H_{2}O_{2}$ levels were significantly decreased in all cell types and remained significantly below baseline in sensory (p≤0.001) and pillar cells (p≤0.01) by 24 h.

**Fig. 4:**
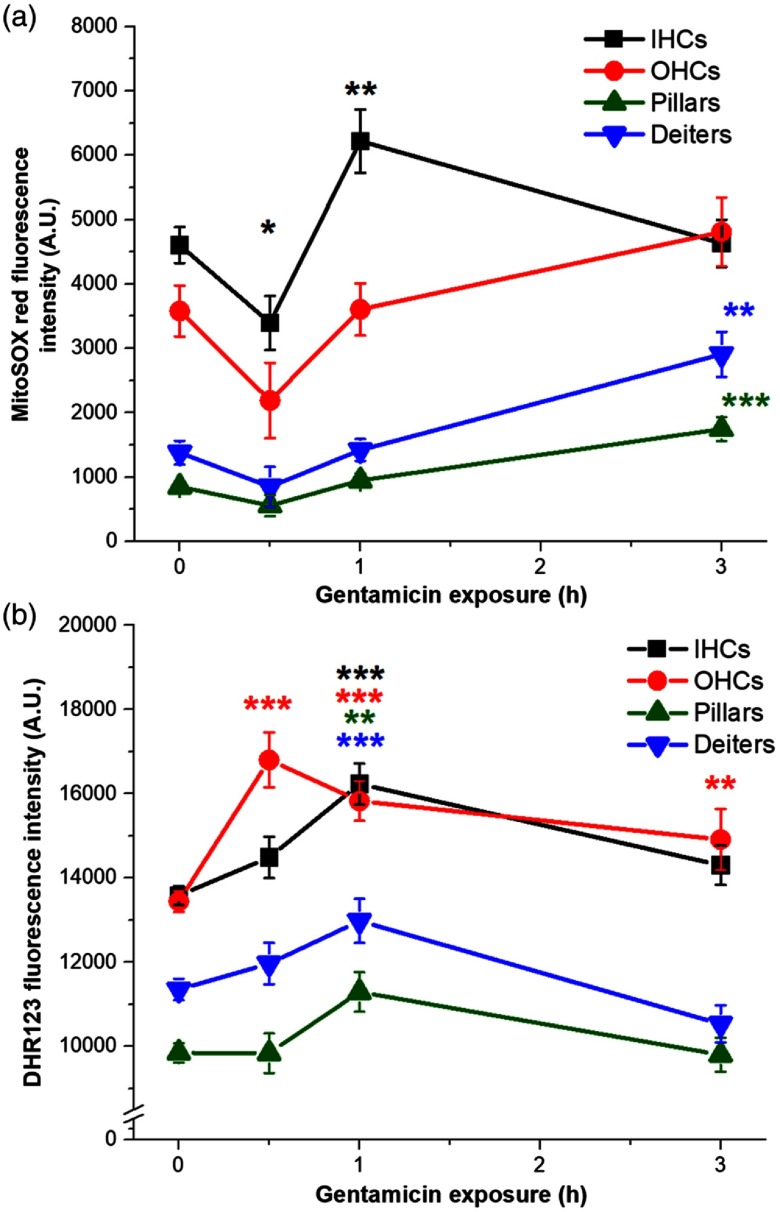
Acute GM exposure increases ${{O_{2}}^{\cdot }}^{-}$ and $H_{2}O_{2}$ levels in sensory and supporting cochlear cells. (a) ${{O_{2}}^{\cdot }}^{-}$ decreased in IHCs at 0.5 h, but rapidly increased at 1 h. By 3 h, ${{O_{2}}^{\cdot }}^{-}$ levels returned to near-baseline levels in sensory cells, but significantly increased in supporting cells. (b) Rapid $H_{2}O_{2}$ increases were seen in OHCs at 0.5 h, in all cells at 1 h and remained elevated in OHCs at 3 h. Error bars = SEM. *p≤0.05, **p≤0.01, ***p≤0.001, relative to baseline.

As ${{O_{2}}^{\cdot }}^{-}$ is a transient ROS and is rapidly dismutated by endogenous antioxidants, we compared ${{O_{2}}^{\cdot }}^{-}$ levels to $H_{2}O_{2}$ production in cochlear sensory and supporting cells during acute GM exposures. Due to the expected rapid conversion, we anticipated that $H_{2}O_{2}$ levels should mirror, or follow very close behind, ${{O_{2}}^{\cdot }}^{-}$ levels. To characterize changes in proximal mitochondrial ROS (${O_{2}}^{\cdot }$) levels, GM-exposed samples were incubated with the mitochondrial-specific superoxide indicator, MitoSOX Red. ${{O_{2}}^{\cdot }}^{-}$ in low- and high-frequency cells and $H_{2}O_{2}$ in low- and high-frequency cells were of similar levels and highly correlated across locations. To assess putative conversion from high-reactive ${{O_{2}}^{\cdot }}^{-}$ to low-reactive, $H_{2}O_{2}$, we calculated the average ${{O_{2}}^{\cdot }}^{-}$ and $H_{2}O_{2}$ from all high- and low-frequency redrived fluorescence intensity measurements for each cell type (Fig. 4). ${{O_{2}}^{\cdot }}^{-}$ levels in IHC initially decreased at 0.5 h (p≤0.05), then increased at 1 h [p≤0.01, Fig. 4(a)]. By 3 h, ${O_{2}^{\cdot }}^{-}$ levels returned to baseline in sensory cells but were significantly increased in pillar (p≤0.001) and Deiters cells [p≤0.01, as shown in Fig. 4(a)]. $H_{2}O_{2}$ levels significantly increased in OHCs at 0.5 h (p≤0.001) and in all cell types at 1 h (p≤0.001). $H_{2}O_{2}$ levels returned to baseline in IHCs and supporting cells at 3 h, but remained elevated in OHCs (p≤0.01). In I/OHCs at 0.5 h, there was a general trend toward decreasing ${{O_{2}}^{\cdot }}^{-}$ and increasing $H_{2}O_{2}$. At 3 h, ${{O_{2}}^{\cdot }}^{-}$ was no longer significantly elevated in OHCs, but $H_{2}O_{2}$ remained elevated.

### 3.3 Endogenous Antioxidants are Altered by GM Exposure

**Fig. 5:**
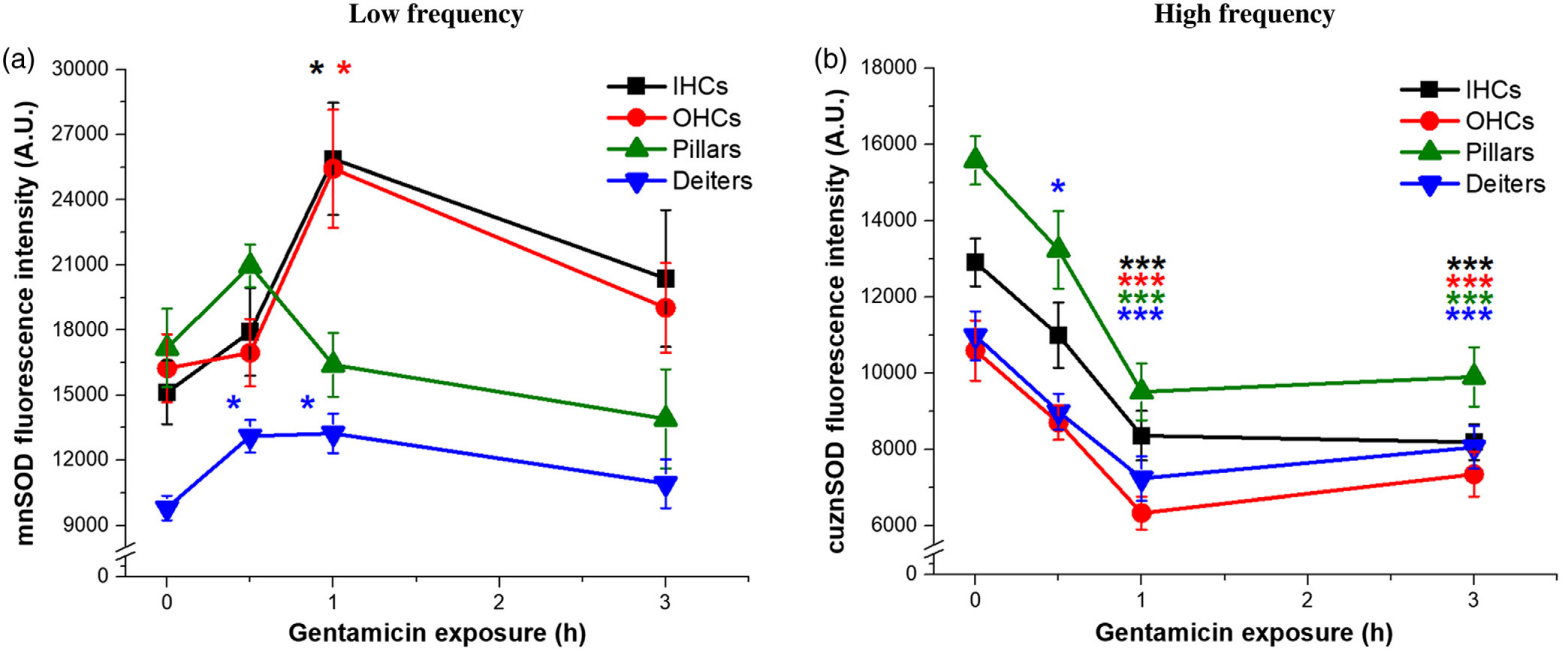
Endogenous antioxidant levels are altered by acute GM exposure. (a) MnSOD, present in the mitochondrial matrix, is transiently, significantly increased in I/OHCs and Deiters cells after 1 h GM exposure. (b) A prolonged and significant decrease in CuZnSOD, present in the cellular cytoplasm, was observed in sensory and supporting cells starting at 1 h of GM exposure. Error bars = SEM. *p≤0.05, ***p≤0.001, relative to baseline.

To determine if increased ${{O_{2}}^{\cdot }}^{-}$ levels and putative conversion to $H_{2}O_{2}$ are associated with antioxidant activity, we assessed SOD levels in GM-exposed cochlea explants. SODs are the first line of defense against cellular ROS, catalyzing the breakdown of ${{O_{2}}^{\cdot }}^{-}$ to $H_{2}O_{2}$ at multiple sites. MnSOD is present in the matrix of mitochondria, whereas CuZnSOD is predominantly present in the cellular cytoplasm. As shown in Fig. 4, data were pooled from low- and high-frequency regions of the cochlea. About 1-h GM exposure significantly increased MnSOD levels in I/OHCs (p≤0.05) and Deiters cells [p≤0.05, Fig. 5(a)]. Notably, MnSOD levels return to baseline levels in all cell types with 3-h exposure [Fig. 5(a)]. Conversely, CuZnSOD significantly decreased in all cell types in the first hour (p≤0.001) and remained significantly below baseline at 3 h (p≤0.001), indicating a difference in endogenous antioxidant responses [Fig. 5(b)].

### 3.4 GM Increases Mitochondrial-Mediated Pro-Apoptotic Signaling and Sensory Cell Loss

The mitochondrial protein, apoptosis-inducing factor (AIF), is a caspase-independent trigger of chromatin condensation, DNA fragmentation, programmed cell death, and is also known to stabilize the mitochondrial ETC complexes. While normally found in the outer mitochondrial membrane, AIF translocates to the cytosol and nucleus when mitochondrial dysfunction is sufficient for signaling irreversible cell death/apoptosis.^36^

**Fig. 6:**
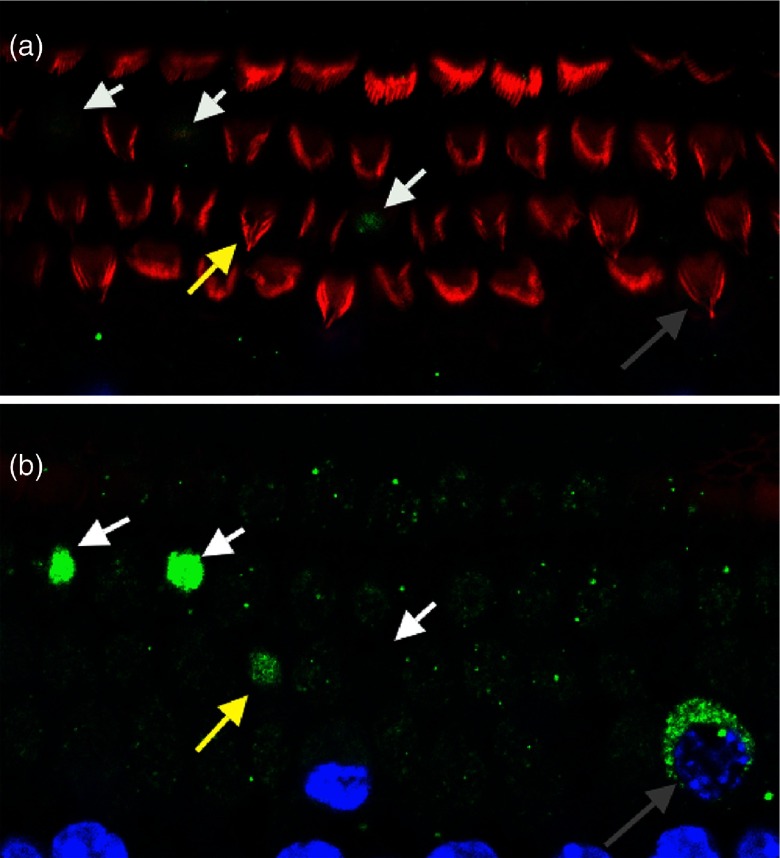
Representative apoptosis-inducing factor (AIF) labeling after acute GM exposure. In cochlear explants, as described in Figs. 1(b) and 1(a) 3-h GM exposure visibly damaged OHC cilia (white arrows), but not IHC cilia (top cell row). AIF fluorescence accumulated in the cytoplasm (yellow, white arrows) of cells with compromised cilia. (b) AIF translocation into the cytoplasm of the same cells can be seen in an image obtained ∼6  μm below the cuticular plate region shown in (a). A cell with AIF accumulating, but not yet condensing, during apoptosis is indicated by a gray arrow. Blue = nuclei, green = AIF, red = F-actin. Arrows indicate identical locations/cells in the same cochlear preparation imaged at different depths.

**Fig. 7:**
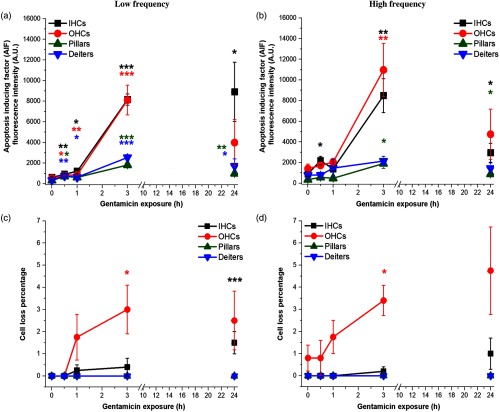
GM exposure increases AIF signaling and sensory cell loss. (a) AIF fluorescence intensity significantly increased in all low-frequency cell types within 0.5 h. A rapid increase in sensory cells occurred 3 h. (b) In high-frequency cells, AIF was significantly increased in IHCs at 0.5 h and rapidly peaked in sensory cells at 3 h. (c) Low-frequency OHC loss significantly increased at 3 h, which is visibly seen in Fig. 6. IHC loss was greatest at 24 h. (d) High-frequency OHC loss significantly increased with 3-h GM exposure. Error bars = SEM. *p≤0.05, **p≤0.01, ***p≤0.001, relative to baseline.

Representative images reveal AIF labeling, as well as varying stages of cellular degeneration prior to apoptosis and ejection from the cochlear epithelium (Fig. 6). Fluorescence intensity measurements were used to quantify the effect of GM on AIF release and subsequent AIF labeling in cochlear cells. AIF fluorescence intensity significantly increased in all low- frequency cell types (IHC, Deiters p≤0.01; OHC, pillars p≤0.05) within 0.5 h of GM exposure [Fig. 7(a)]. AIF levels further increased at 1 h (IHCs, Deiters p≤0.05; OHCs p≤0.01) and 3 h (all cell types, p≤0.001), and remained elevated at 24 h in IHCs (p≤0.05), pillars (p≤0.01), and Deiters cells (p≤0.05). In the high-frequency region, AIF was transiently, significantly increased in IHCs at 0.5 h (p≤0.05). By 3 h, AIF was significantly increased in I/OHCs (p≤0.01) and pillar cells (p≤0.05). AIF remained elevated in IHCs and pillar cells at 24 h (p≤0.05) and returned to baseline in OHCs and Deiters cells [Fig. 7(b)]. The magnitude of AIF intensity changes was distinctly higher in I/OHCs, though the same trends are apparent in both sensory and supporting cells.

As shown in Figs. 6 and 7, severe insult, indicated by increased AIF fluorescence intensities, is associated with cochlear cell ejection. We calculated the fraction of ejected cells in GM-exposed, AIF-labeled samples. For low-frequency cochlear cells, significant OHC-specific loss occurred at 3 h (p≤0.05), while significant IHC loss occurred after 24 h (p≤0.001). High-frequency OHC loss was also seen at 3 h (p≤0.05), but no significant loss was seen in high-frequency IHCs. Significant cell loss was not observed in low- or high-frequency supporting cells during 24 h GM exposures.

In GM-exposed samples, condensing apoptotic cells missing apical specializations, including the cuticular plate and cilia, were observed (white arrows, Fig. 6). Condensing apoptotic cells with the cuticular plate still intact were also observed [yellow arrows, Fig. 6(a)]. Additionally, cells with AIF-labeled cytoplasm that had not begun nuclear condensation were observed [gray arrow, Fig. 6(b)]. Finally, we observed missing sensory cells, indicating that insult had progressed to the point of cellular ejection [Fig. 6(b)].

### 3.5 GM Pretreatment Increases RTN-Induced ${{O_{2}}^{\cdot }}^{-}$ in IHCs While Inhibiting RTN-Induced ${{O_{2}}^{\cdot }}^{-}$ in OHCs

**Fig. 8:**
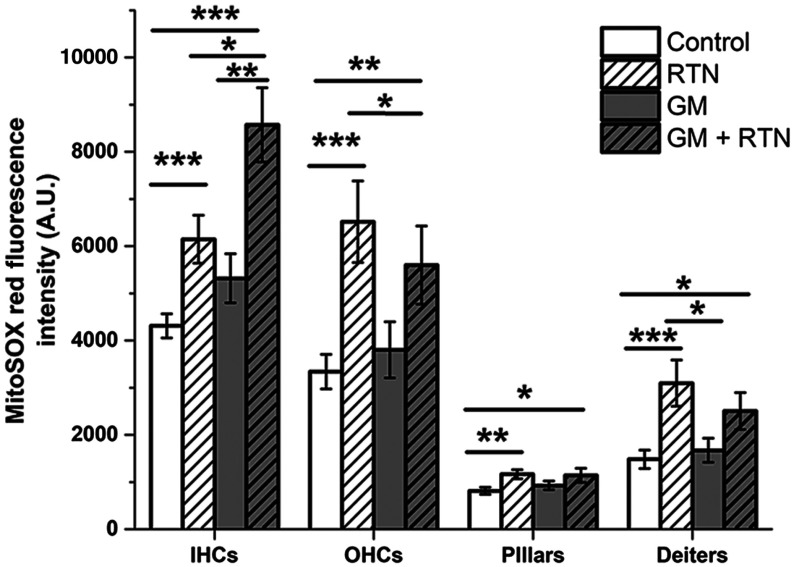
GM pretreatment increases RTN-induced ROS in IHCs while inhibiting RTN-induced ROS in OHCs. RTN increases ${{O_{2}}^{\cdot }}^{-}$ relative to baseline in all cell types. In IHCs, 1 h GM + RTN produces significantly greater increases in ${{O_{2}}^{\cdot }}^{-}$ than RTN and GM alone. In OHCs and supporting cells, GM+RTN shows a nonsignificant trend toward decreased ${{O_{2}}^{\cdot }}^{-}$ when compared with RTN alone.

As previously shown, GM exposure caused significant increases in ROS levels and corresponding changes in both endogenous metabolism and antioxidant systems within 1 h. However, these data do not indicate the mechanism(s) by which GM induces metabolic changes and subsequent downstream effects, including sensory cell loss. Due to the increases seen in ${{O_{2}}^{\cdot }}^{-}$ levels, we assessed the role of complex I as a primary site of ${{O_{2}}^{\cdot }}^{-}$ production during GM exposure. We used the inhibitor rotenone (RTN) to block the transfer of electrons from complex I iron–sulfur centers to ubiquinone in the electron transport chain (ETC) that results in complex I-specific ROS production. If GM inhibits complex I in a similar manner, we would anticipate comparable increases in ROS production. As expected, RTN alone significantly increased ${{O_{2}}^{\cdot }}^{-}$ levels in all cell types, relative to baseline levels (Fig. 8). These measurements were compared with cochlea exposed only to GM for 1 h. In IHCs, GM alone increased ${{O_{2}}^{\cdot }}^{-}$ (p=0.054) and GM + RTN together significantly increased ${{O_{2}}^{\cdot }}^{-}$ levels compared with GM or RTN alone. However, the combined effects of GM and RTN did not significantly increase ${{O_{2}}^{\cdot }}^{-}$ relative to GM or RTN alone in OHCs or supporting cells, suggesting both a nonadditive effect of GM and RTN and fundamental differences in ${{O_{2}}^{\cdot }}^{-}$ production and/or accumulation across cell types (Fig. 8).

## 4 Discussion

**Fig. 9:**
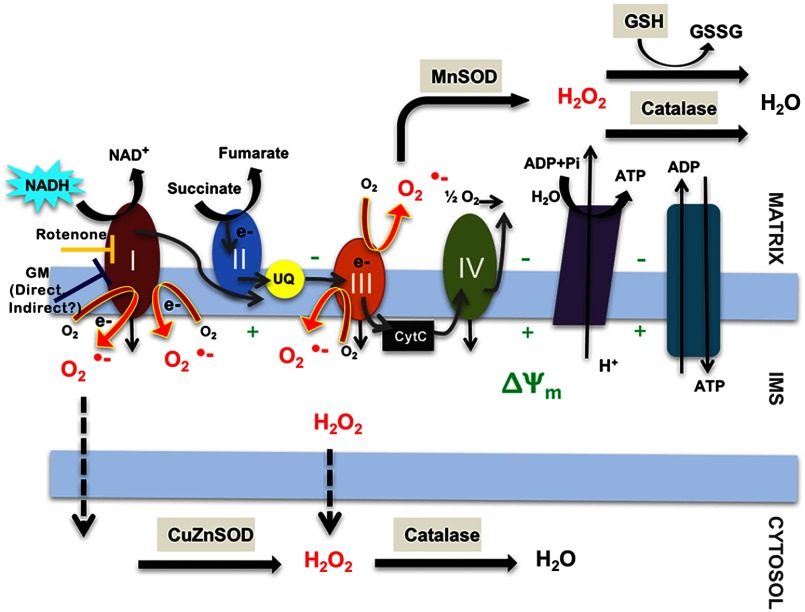
ROSs are a natural byproduct of the electron transport chain. Mitochondria produce low levels of ROS, mainly ${{O_{2}}^{\cdot }}^{-}$ during normal metabolism. When ${{O_{2}}^{\cdot }}^{-}$ is released into the matrix, it is rapidly converted to $H_{2}O_{2}$ by MnSOD. If ${{O_{2}}^{\cdot }}^{-}$ leaks into the intermembrane space, they may escape to the cytosol, where they can be converted to $H_{2}O_{2}$ by CuZnSOD. $H_{2}O_{2}$ is further broken down into water by catalase and glutathione in the cytoplasm.

Irreversible cochlear cell death remains a costly side effect of AG use. Understanding the mechanism(s) of AG-induced cochlear cell loss is integral to combating the side effects of these life-saving antibiotics. Although many studies have focused on the long-term (≥24  h) effects of AG exposure, NADH fluorescence lifetime imaging performed by our research group using the same GM dose and similar time points indicated metabolic perturbations occur within the first 0.5 h of AG exposure.^22^ GM exposure increases NAD(P)H lifetime and intensity in low- and high-frequency sensory cells, indicating metabolic changes occur within this timeframe. Though the mechanism(s) of AG-induced insult have yet to be determined, ROS are likely key players. ROS have long been shown to form in cochlear sensory cells following AG exposure.^17^^,^^37^^,^^38^ While cochlear cells have intrinsic antioxidant pathways to combat normal ROS production, considerable ROS increases are likely to overwhelm these systems and drive cells toward apoptosis. Therefore, we extended NADH intensity imaging using a variety of fluorescence probes to investigate and correlate downstream changes in ROS accumulation, mitochondrial function, and cellular viability. A model of the potential inter-relationship between all of these factors is shown in Fig. 9. The current studies were designed to further our understanding of acute (≤24  h) AG-induced metabolic dysfunction, including increased ROS production, and the resulting cellular demise.

### 4.1 Significant Metabolic Changes Occur with 0.5 h GM Exposure

Fluorescence intensity imaging during acute GM exposure revealed rapid changes in NADH levels and MMP. As previously shown, GM accumulates selectively in cochlear sensory cells in under 0.5 h.^22^ By the first hour of GM exposure, we saw significant NADH increases in low- and high-frequency sensory and supporting cells. By 12 h, these effects had diminished and NADH levels remained near baseline up to 24 h later [Figs. 3(a) and 3(b)]. The protonmotive force across the mitochondria consists of a pH and electrical gradient (MMP). TMRE was used to detect MMP changes across the inner mitochondrial membrane (Fig. 9). Although it is not a measure of the proton gradient, increases in TMRE fluorescence intensity indicate hyperpolarization of the inner mitochondrial membrane.^39^ As shown in Figs. 3(c) and 3(d), maximal MMP changes (hyperpolarization) occur within 0.5 h of GM exposure, followed by a significant decrease in MMP by 1 h. When the normal action of complex V/ATP synthase is reversed, ATP is brought into the mitochondria through the adenosine nucleotide transporter (ANT) and ATP is hydrolyzed to produce more substrate ADP and re-establish the proton gradient across the inner membrane (Fig. 9). This occurs when metabolism has been severely disrupted and results in hyperpolarization.^40^ Hyperpolarization is a strong indicator of metabolic perturbation and has been proposed to be the “point of no return” in apoptotic signaling.^41^^,^^42^ Interestingly, the greatest MMP increases are seen in high-frequency OHCs and Deiters cells; this correlates with previous observations that high-frequency OHCs are the first to respond to metabolic perturbations.^20^^,^^43^^–^^45^ Drastic MMP changes indicate reduced electron transport fidelity, thereby increasing the likelihood that electrons form ROS byproducts.

### 4.2 ROS Levels Change Rapidly with Acute GM Exposure

Increased ROS are a consequence of metabolic perturbations, particularly in the mitochondria, and appear to be a key player in hair cell death.^26^^,^^46^ Complex I is a major site of mitochondrial ${{O_{2}}^{\cdot }}^{-}$ production (Fig. 9), and complex I-specific ROS may increase by two mechanisms: either when the NADH/NAD+ ratio is high, or when electron donation to coenzyme Q is coupled with a high MMP, leading to reverse electron transport (RET).^47^ Rotenone increases ${{O_{2}}^{\cdot }}^{-}$ production at complex I (Fig. 9) by backing up electrons onto flavin mononucleotide (FMN). RET occurs when the CoQ pool is reduced, increasing the protonmotive force (Δp) and forcing electrons back to complex I. This causes NAD+ to be reduced and form NADH, increasing the probability of ${{O_{2}}^{\cdot }}^{-}$ formation (Fig. 9). Thus, the use of inhibitors such as RTN also abolishes excess ${{O_{2}}^{\cdot }}^{-}$ production from RET. It is also noted that RET-associated ${O_{2}^{\cdot }}^{-}$ production is heavily dependent on Δp and a small Δp decrease will lead to near-complete elimination of RET.

Because of the rapid MMP elevations seen in Figs. 3(c) and 3(d), RET is unlikely to be responsible for the ${{O_{2}}^{\cdot }}^{-}$ increase seen 1-h post GM-exposure. Complex I ${{O_{2}}^{\cdot }}^{-}$ production is also thought to be more sensitive to the pH component of Δp than to the MMP component. NADH peaks at 1 h, the same time that the largest ${{O_{2}}^{\cdot }}^{-}$ increases are seen. Though complex III can also produce ${{O_{2}}^{\cdot }}^{-}$ (particularly in the presence of inhibitors such as antimycin), the physiological amount produced is far lower than the maximum achieved by complex I.^47^

Complex-I deficiencies and inhibition have been associated with both significant MMP depolarization and hyperpolarization; the direction of perturbation appears to be cell-specific and dependent upon inhibitor concentration and duration of exposure.^40^ Notably, IHCs were also the only cell type to produce significantly more ${{O_{2}}^{\cdot }}^{-}$ with 1-h GM exposure, whereas OHC did not show a significant ${{O_{2}}^{\cdot }}^{-}$ with acute exposure and supporting cells had increases ${{O_{2}}^{\cdot }}^{-}$ levels by 3 h [Fig. 4(a)]. At 0.5-h GM exposure, MMP reached its maximum hyperpolarized state; at this same point, ${{O_{2}}^{\cdot }}^{-}$ levels decreased in all cell types. MMP hyperpolarization increases the energy required to pump protons across the membrane and maintain electron flow through the ETC. The decrease in ${{O_{2}}^{\cdot }}^{-}$ levels at 0.5 h, therefore, may be a direct result of MMP perturbations.

### 4.3 Endogenous Antioxidants are Differentially Affected by GM Exposure

The ETC, located in the inner mitochondrial membrane (IMM), has been well established as the main site of mitochondrial ${{O_{2}}^{\cdot }}^{-}$ production, particularly at complexes I and III, respectively. Complex I (NADH dehydrogenase) catalyzes electron transfer to ubiquinone (CoQ). Electrons that do not bind to CoQ due to some form of ETC inhibition may form ${{O_{2}}^{\cdot }}^{-}$ at complex I (Fig. 9). Complex III (cytochrome c reductase) has two reaction centers, the ubiquinol-oxidation ($Q_{o}$) and ubiquinone-reduction ($Q_{i}$) sites. Electron exchange during the Q-cycle may result in leak and generation of ${{O_{2}}^{\cdot }}^{-}$ at either site.^48^ ${{O_{2}}^{\cdot }}^{-}$ formed by complex I and the $Q_{o}$ site of complex III are produced in the inner membrane space (IMS); from here, they may leak into the cytosol, where molecules can be dismutated to $H_{2}O_{2}$ by CuZnSOD, then converted to water by catalase (Fig. 9). ${{O_{2}}^{\cdot }}^{-}$ molecules formed at the $Q_{i}$ site or those that leak through the IMS remain in the matrix until they are converted to $H_{2}O_{2}$ by MnSOD. Peroxides are converted to water either by glutathione or catalase pathways (Fig. 9).

We assessed ${{O_{2}}^{\cdot }}^{-}$ and $H_{2}O_{2}$ levels with acute GM exposure as downstream effects of significant metabolic changes that occur within the first 24 h. While significant ${{O_{2}}^{\cdot }}^{-}$ changes were seen in IHCs within the first hour, these changes did not persist for the full 3 h [Fig. 4(a)]. This is likely due to the rapid conversion of ${{O_{2}}^{\cdot }}^{-}$ to $H_{2}O_{2}$ by MnSOD and CuZnSOD in the matrix and cytosol (Fig. 9). Furthermore, the lack of increased ${{O_{2}}^{\cdot }}^{-}$ in OHCs suggests fundamental differences in ROS mitigation between cell types. About 3-h GM exposure was needed to significantly increase ${{O_{2}}^{\cdot }}^{-}$ levels in supporting cells, likely attributable to slower uptake due to the lack of specialized mechanotransduction channels. $H_{2}O_{2}$ significantly increased in all cell types at 1 h, and this increase persisted in OHCs up to 3 h [Fig. 4(b)]. The rapid decrease in $H_{2}O_{2}$ may indicate that secondary antioxidant pathways, including glutathione, have been stimulated for $H_{2}O_{2}$ removal.

The persistence of increased $H_{2}O_{2}$ in OHCs also indicates fundamental differences between different cell types’ ability to effectively remove $H_{2}O_{2}$. To characterize SOD activity during GM exposure and subsequent ROS increases, we looked at matrix (SOD2, Mn) and cytoplasmic/IMS (SOD1, CuZn) SOD levels. We found that GM significantly increased $H_{2}O_{2}$ levels in all cell types with 1 h of exposure. At the same time point and concentration, GM most significantly decreased CuZnSOD levels in all cell types. There are fundamental differences between these enzymes that affect their ability to moderate ${{O_{2}}^{\cdot }}^{-}$ levels. CuZnSOD activity decreases under several conditions, including radiation, aging, and catalase inhibition. Additionally, *in vitro* $H_{2}O_{2}$ exposure has been shown to inactivate human CuZnSOD by destroying the enzyme’s active site at physiologically relevant conditions (pH 7.4, 37°C).^49^ SOD1 is known to be inactivated by excess product formation ($H_{2}O_{2}$) and has a half-life of 6 to 10 min, compared with SOD2/MnSOD’s half-life of 5 to 6 h.^50^ Further, exposure to $H_{2}O_{2}$ without the presence of ${O_{2}^{\cdot }}^{-}$ does not appear to deactivate CuZnSOD, and MnSOD also appears to help prevent CuZnSOD deactivation (*in vitro*).^49^ In the studies presented here, we observed an increase in MnSOD within 1 h of GM exposure and a decrease in CuZnSOD at the same timepoint (Fig. 5). Though MnSOD is not inhibited by $H_{2}O_{2}$, this could imply that sufficient ${{O_{2}}^{\cdot }}^{-}$ and $H_{2}O_{2}$ have been produced to reduce CuZnSOD levels, but MnSOD cannot effectively protect CuZnSOD. The decrease in CuZnSOD and return of MnSOD to baseline levels may indicate that secondary antioxidant systems such as glutathione and catalase are responding to the metabolic perturbations and increased $H_{2}O_{2}$ levels.

### 4.4 GM’s Mechanism of Action Varies between Cell Types

We assessed ${{O_{2}}^{\cdot }}^{-}$ production when GM and RTN were applied together to study the contribution of complex I to overall ROS production. Their respective mechanisms of action could act independently, inhibit, or exacerbate one another. GM alone does not increase mitochondrial ${{O_{2}}^{\cdot }}^{-}$ levels to the extent that RTN alone does in any cell type (Fig. 8, solid gray). In IHCs, GM+RTN induced significantly higher ${{O_{2}}^{\cdot }}^{-}$ levels than RTN or GM alone, suggesting that GM exacerbates ${{O_{2}}^{\cdot }}^{-}$ production in IHCs. Because RTN irreversibly blocks electron donation by complex I, however, this would imply that GM induces ROS formation elsewhere in the mitochondria or significantly increases complex I electron flow at 1 h. This data may, therefore, indicate that GM induces ${{O_{2}}^{\cdot }}^{-}$ increases at locations besides complex I, which has a summative effect in IHCs when observed with RTN-induced ${{O_{2}}^{\cdot }}^{-}$.

GM+RTN-induced ${{O_{2}}^{\cdot }}^{-}$ levels in OHCs and supporting cells were higher than GM alone but lower than ${{O_{2}}^{\cdot }}^{-}$ levels produced by RTN alone. This suggests that GM and RTN inhibit each other’s effects in these cell types. Differences in the cell types’ responses may indicate varying electron flow through complex I and/or may also be attributed to fundamental metabolic or antioxidant differences between cell types.

### 4.5 GM Drives Cochlear Cells Toward Apoptotic Pathways

As shown in Fig. 6, acute GM exposure induces morphological changes consistent with pro-apoptotic signatures. Different apoptotic processes have been proposed as the mechanism of cochlear cell death. AIF is a caspase-independent pathway that has been suggested to be activated in damaged cochlear cells.^46^ Upon postouter mitochondrial membrane permeabilization, AIF translocates through the cytoplasm to the nucleus, where it causes DNA fragmentation and chromatin condensation. It is thought to affect oxidative phosphorylation through both redox activity and direct assembly or stability of the respiratory complexes.^36^ Because of its potential role in redox metabolism signaling, we investigated its expression during GM exposure as a marker of apoptosis.^51^

As shown in Fig. 3, we observed significant shifts in NADH levels with acute GM exposure. These metabolic changes may interrupt AIF’s normal function in mitochondrial metabolism in addition to activating the apoptotic pathway. We also observed the percent of ejected cells is highest in OHCs and that significant OHC loss occurs ∼9  h before IHC loss. OHCs have been found to respond more quickly to AG-induced insult, coinciding with the larger fraction of ejected cells (Fig. 7).

**Fig. 10:**
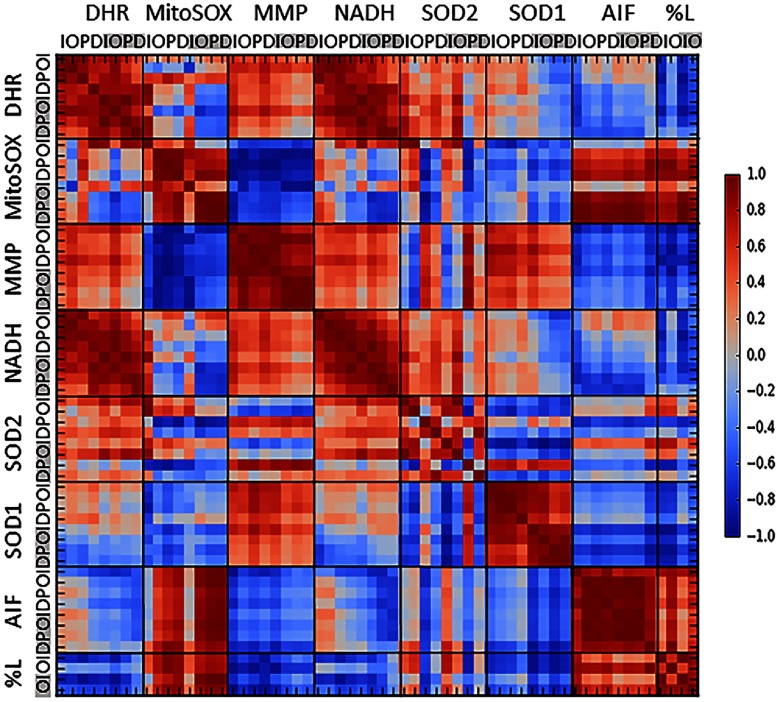
Correlated changes in mitochondrial metabolism, ROS accumulation and cellular demise in cochlear cells exposed to acute GM. Pearson’s pairwise correlation coefficients were calculated between all time course measurements (DHR, MitoSOX, NADH, SOD1, SOD2, AIF, and % cell loss), all cell types (I, IHC; O, OHC; P, pillar; D, Deiters), and both cochlear locations (Apex not highlighted and base highlighted in gray). Red = positive linear correlation, white = no correlation, blue = anticorrelation.

To verify associations between metabolic dysfunction (NADH, MMP), ROS production (DHR123, MitoSOX labeling), and cochlear cell viability (AIF labeling, percent cell loss), during acute GM exposures, correlations were performed across all time course measurements obtained from high- and low-frequency sensory and supporting cells (Fig. 10). Across all cell types (IHCs, OHCs, pillars, and Deiters), measurements of metabolic dysfunction (changes in NADH, MMP) trended toward a positive correlation with each other. Consistent with emerging evidence indicating acute GM rapidly alters mitochondrial function resulting in excess ROS production, $H_{2}O_{2}$ production throughout the acute GM exposure period was positively correlated with changes in NADH and MMP in all locations and cell types. On the other hand, ${{O_{2}}^{\cdot }}^{-}$ production was consistently anticorrelated with MMP and, to a lesser extent, with NADH in all locations and cell types. This anticorrelation is consistent with the conversion of highly reactive ${{O_{2}}^{\cdot }}^{-}$ to $H_{2}O_{2}$ through endogenous antioxidants, such as MnSOD and CuZnSOD. Indeed, changes in ${{O_{2}}^{\cdot }}^{-}$ accumulation were positively correlated with increases in mitochondria-specific antioxidant, MnSOD and anti-correlated with the short half-life (6 to 10 min), cytoplasmic antioxidant, CuZnSOD. Notably, as shown in Fig. 9, additional endogenous antioxidants are likely involved in converting GM-induced ${{O_{2}}^{\cdot }}^{-}$ to $H_{2}O_{2}$. Finally, although high-reactive ${{O_{2}}^{\cdot }}^{-}$ appears to be rapidly converted to low-reactive $H_{2}O_{2}$ in cochlear sensory and supporting cells, increases in AIF and cell loss were positively correlated with ${{O_{2}}^{\cdot }}^{-}$ accumulation while largely anticorrelated with $H_{2}O_{2}$ accumulation. Together, the aforementioned data and associated correlations indicate acute GM triggers mitochondrial dysfunction and excessive production and accumulation of ROS resulting in cochlear cell death.

## 5 Conclusion

Given its clinical prevalence and associated studies by our group and others, we selected GM as a representative AG for these studies. It is, however, important to acknowledge that as a class, AG antibiotics show varying degrees cochlear and vestibular cell toxicities.^3^^,^^25^ While all AG’s appear to bind to mitochondrial ribosomal RNA (rRNA) resulting in changes in recognition and selection of transfer RNA (tRNA) resulting in deficits in mitochondrial ribosomal translation and translocation, rRNA binding affinities are reported to vary across AGs.^52^^–^^54^ Furthermore, structural differences across AG’s may contribute to such differences in ototoxicity.^25^^,^^55^^,^^56^ Regardless of the subtleties of AG-specific alterations in mitochondrial dysfunction, similar ribosomal sites and downstream translation and translocation effects occur across AGs. The current studies correlate changes in metabolic function, ROS production, and cochlear cell demise in response to the clinically prevalent AG, GM. Although additional studies are required, given similar ribosomal binding and modifications across AGs, the correlations described herein are predicted to be similar across all nine AGs currently in clinical use.

Fluorescence intensity imaging revealed rapid metabolic changes and resulting downstream effects in cochlear cells. Following MMP hyperpolarization, ROS levels increased, which was correlated with changes in antioxidant levels. This was followed by increased apoptosis and cellular ejection. These experiments were designed to better understand the consequences of AG-induced metabolic changes in cochlear sensory and supporting cells.

Our results further support these findings, showing a difference between metabolic changes in high- and low-frequency regions of the cochlea as well as ROS production in cochlear cell types. Further, AG’s mechanism of action and subsequent mitigation of ROS may differ between cell types. The results of these studies support an overall mechanism that likely contributes to AG-induced as well as age-related and noise-induced hearing loss. The mitochondrion is further implicated as a key player in the mechanism, including ROS production and resulting downstream effects.
